# Hearing assessment in Pomeranian dogs with and without Chiari-like malformation based on brainstem auditory evoked response wave latencies

**DOI:** 10.3389/fvets.2026.1863681

**Published:** 2026-06-26

**Authors:** Marta Płonek, Rachel Malkani, Annabel Bakker, Vivianne Stoute, Larissa Hoogervorst-Spek, Paul J. J. Mandigers

**Affiliations:** 1 Evidensia Referral Hospital Arnhem, Arnhem, Netherlands; 2 IVC Evidensia, Bristol, United Kingdom; 3 Department of Clinical Sciences, Faculty of Veterinary Medicine, University of Utrecht, Utrecht, Netherlands

**Keywords:** brainstem auditory evoked response, wave latency, Chiari malformation, Pomeranian, hearing

## Abstract

**Introduction:**

Chiari-like malformation (CM) has been associated with hearing abnormalities in humans and Cavalier King Charles Spaniels, but hearing function has not previously been assessed in Pomeranians with CM. This pilot study evaluated whether Pomeranians with predominantly mild CM exhibit abnormalities in brainstem auditory evoked responses (BAER).

**Materials and methods:**

Forty-two Pomeranian dogs underwent MRI and BAER testing. Dogs were classified according to CM severity based on MRI findings. Thirty-two dogs were classified as CM affected (31 CM1, 1 CM2), and 10 dogs served as CM0 controls. Suprathreshold BAER recordings were obtained under general anaesthesia at intensities from 20 to 90 decibels normalised hearing level (dBnHL). Absolute latencies of waves I, III and V, and interpeak intervals I–III, III–V and I–V were measured for both ears. Reference ranges were established from control dogs, and a linear mixed-effects model was used to assess the effects of group and ear side on latency values.

**Results:**

No significant differences were identified between CM and control dogs for any BAER wave latency or interpeak interval. There was substantial overlap between groups across all stimulus intensities. However, a significant ear effect was observed, with the right ear showing slightly longer latencies than the left ear (estimate = 0.047, *t* = 3.67, *p* < 0.001).

**Conclusion:**

These findings suggest that mild CM in Pomeranians does not result in measurable abnormalities in BAER. Further studies, including a larger number of dogs with moderate or severe CM, particularly CM2, and incorporating morphometric analyses and BAER amplitude measurements, are warranted.

## 1 Introduction

Chiari-like malformation (CM) is a developmental disorder of the caudal cranial fossa described both in humans and animals (1). Canine CM is characterised by a decreased volume of the caudal fossa and caudal displacement of the caudal cerebellar vermis into or through the foramen magnum and increased cerebellar volume and is often seen in combination with syringomyelia (SM) (1–3). In veterinary medicine it is usually observed in small (brachycephalic) dog breeds and toy breeds, such as the Chihuahua, Griffon, Affenpinscher, and Pomeranian. One of the possible causes could be brachycephalism, although there are brachycephalic breeds such as the French bulldog without CM but suffering from SM and dolichocephalic breeds with both CM and SM (4–8).

The clinical signs of CM vary depending on its severity and progression. In humans, they range from headaches, blurred vision, hearing abnormalities to dizziness, and, in advanced cases, difficulty walking and bladder dysfunction (9). In children, CM has been reported to be associated with sensorineural hearing loss affecting one or both ears, to various extent (10–12). In dogs, they may include phantom scratching, head scratching, air licking, sensitivity around the neck (both provoked and unprovoked) and in more advanced cases, spontaneous signs of pain, cranial nerve and proprioceptive deficits as well as signs of general discomfort (13).

Brainstem auditory evoked responses (BAER), used to assess the functional integrity of the auditory pathway, have been reported to be abnormal in human patients with CM. These abnormalities included prolonged wave V and wave I-V inter-peak latencies as well as the absence of wave III (14, 15). One study looking at BAER waveforms in a cohort of Cavalier King Charles Spaniels with varying degrees of CM (9 dogs with CM 1 and 11 dogs with CM2, without a CM0 control group) found morphologic changes in all tested dogs including variable BAER waveform morphology and longer latencies in CM2 dogs for selected waves and stimulus intensities, although the effects were not consistent across all BAER parameters (16).

Several theories have been proposed to account for hearing abnormalities in humans with CM. These include traction on the vestibulocochlear nerve due to brainstem herniation, compression of the cochlear nuclei and/or the vestibulochochlear nerve by herniated cerebellar tonsils and vascular compromise caused by impaired microcirculation of the posterior inferior cerebellar artery (10, 14, 17). In addition, cochlear damage may occur as a result of increased CSF pressure through an anomalous cochlear aqueduct. It is unclear whether the mechanisms described in humans apply to dogs with CM.

To date, no studies have been performed assessing hearing function on Pomeranians with CM. A recent study revealed that up to 50% of all Pomeranians suffer from CM ((18)). Investigating potential functional consequences, including auditory ones, is clinically relevant. If CM is associated with altered auditory pathway conduction, this could contribute to relevant signs or behavioural changes and may influence the interpretation of hearing assessments in this breed. Therefore, evaluating BAER parameters in Pomeranians with and without CM may help clarify whether this common morphological abnormality is associated with measurable auditory pathway dysfunction. The aim of this study is to determine whether Pomeranian dogs with CM exhibit BAER abnormalities similar to those observed in CKCS and in human patients with the condition.

## 2 Materials and methods

Forty-two dogs without a history of hearing abnormalities were sedated and underwent MRI and CT imaging for CM/SM grading and brainstem auditory evoked response (BAER) recording from January 2022 to July 2025. Owners and dogs could participate only if written informed consent was obtained from the owner prior to inclusion. Before starting this study, approval was sought from the animal welfare body at Utrecht University. Although approval was not required, as all dogs included were referred for clinical reasons, the complete procedure was evaluated by the animal welfare body, and approved and registered under number 16205-20-26. The imaging studies were performed under general anaesthesia (with individualised anaesthetic protocols) in sternal recumbency using a high-field MRI scanner (1.5 T MRI, Canon Vantage Elan, Otawara-shi, Japan) and a 16-slice CT scanner (Siemens SOMATOM.go, The Netherlands).

All MR images were re-evaluated using the Horos DICOM viewer graphical software by the last author (PJJM), an ECVN board-certified diplomat. All scans were evaluated using the BVA/KC scheme^1^ with two important adaptations.

CM was classified according to the grading system described in previous studies (19).

- CM0—No cerebellar herniation or impaction (cerebellar uvula rostral to foramen magnum).
- CM1—Cerebellar impaction (cerebellar uvula on the line of the foramen magnum, no CSF present dorsal to the cervicomedullary junction) and non-rounded shape.
- CM2—Cerebellar herniation (cerebellar uvula caudal to the line of the foramen magnum, no CSF present dorsal to the cervicomedullary junction).
- CM3—Cerebellar herniation resembling CM2, but the posterior part of the cerebellum is herniated and shaped like a tongue.
- CM4—Cerebellar herniation resembling CM2, but the posterior part of the cerebellum is severely herniated and distinctly tongue-shaped.

**Figure 1:**
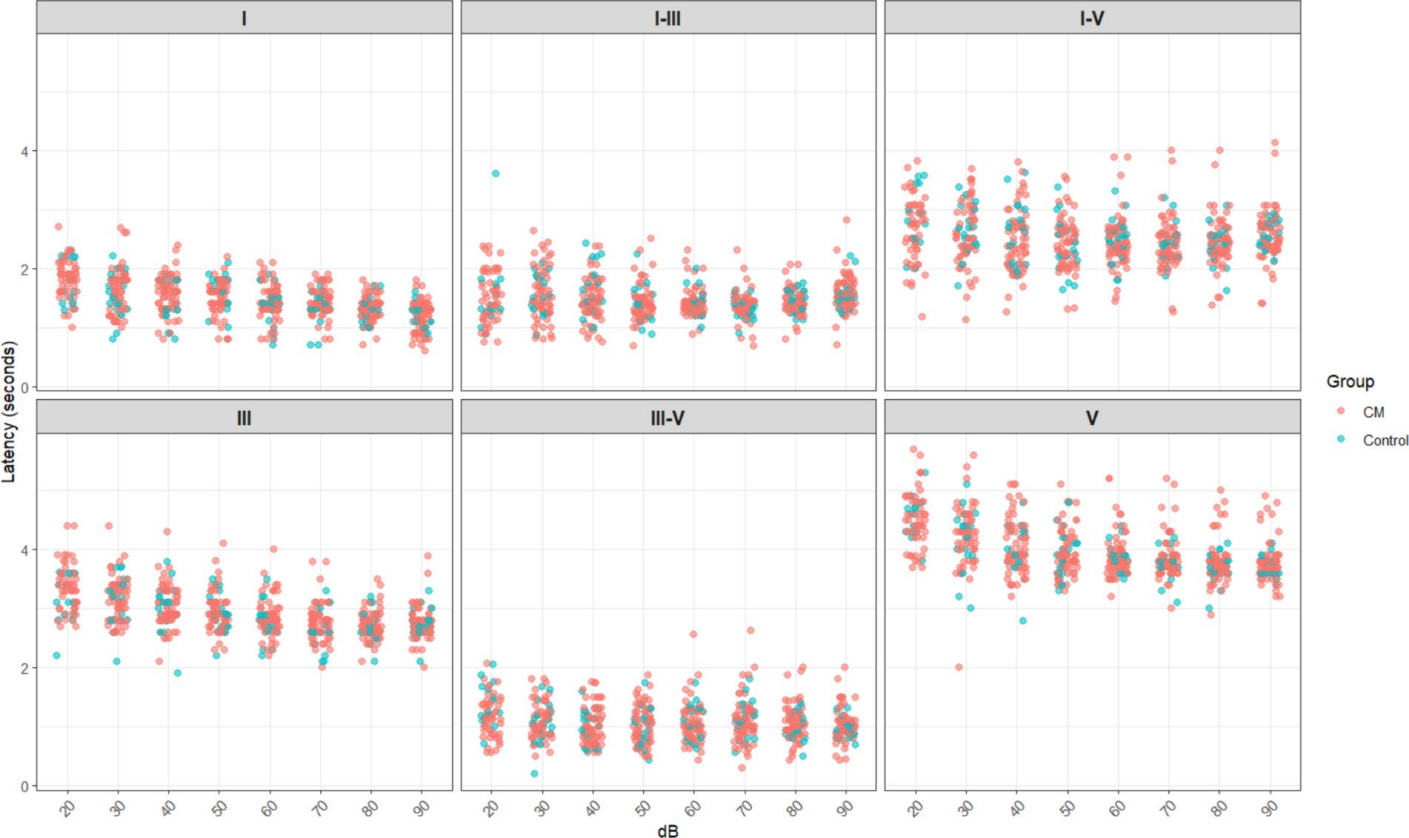
Scatterplots of BAER absolute latencies (waves I, III, and V) and interpeak latencies (I–III, III–V, and I–V) across stimulus intensities from 20 to 90 dB in dogs with CM and controls. Each point represents an individual ear. There was substantial overlap between CM and control groups across all measurements and intensities, with no obvious group-related differences.

The dogs were allocated into the different CM grading groups (14). Dogs affected with CM were pooled into the CM group. In addition, the MRI images were evaluated to confirm normal anatomy of the external, middle, and inner ear. Only dogs with imaging findings consistent with normal structures in these regions were included in the study (Figure 1).

The BAER study was performed following general anesthesia after MRI using a Cubaudio (Path Medical GmBH, Germany) portable device (Ref. 100260-CUB) with insert earphones (single-use 10 mm foam eartips—Etymotic Research). BAER was recorded by placing stainless steel subdermal needle electrodes at the tragus of the tested ear, the non-inverting electrode at the vertex and the ground electrode at the neck. This montage has been previously described using the same device (20).

**Figure 2:**
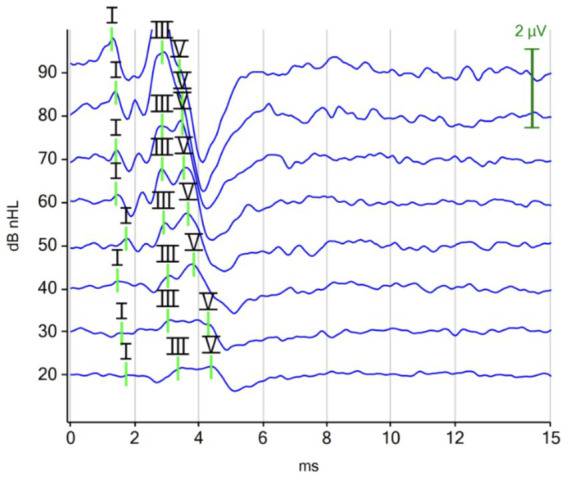
A BAER recording of the left ear in one of the control dogs at stimulus intensities from 20 to 90 dB nHL. Peaks I, III, and V are indicated for each trace where identifiable. The *x*-axis represents time in milliseconds (ms), and the *y*-axis represents stimulus intensity in decibels normal hearing level (dB nHL).

The BAER were recorded using alternating click stimulation (30 Hz) at alternating polarity with a stimulus duration of 0.1 ms, and a decreasing stimulus intensity from 90 dBnHL to 20 dBnHL was recorded for both ears, using a masking noise that was 30 dBnHL below stimulation intensity of the recorded ear. The recordings for each signal intensity for each ear were averaged (6,000). Once the BAER recordings were obtained, the absolute latencies of waves I, III, and V were measured, and interpeak intervals (I–III, I–V, III–V) were calculated using the Mira Software (ver. 2.4.5.9510). Only BAER traces with clearly identifiable and reproducible waveforms were included in the analysis (Figure 2). Traces with poorly defined or unrecognizable peaks were excluded.

The data of the BAER recordings was placed an Excel spreadsheet, with each row representing a single waveform measurement. Variables included dog identification, study group (CM or control), BAER wave (I, III, or V), latency in milliseconds, ear side (left or right), age, sex, and stimulus intensity (dB nHL). Separate rows were entered for each wave, ear, and stimulus intensiy combination.

To establish baseline data, BAERs were also recorded in 10 healthy control Pomeranian dogs. Each dog contributed paired ear measurements per intensity (left ear, right ear). For each wave latency and interpeak interval, descriptive statistics were calculated, including the mean ± standard deviation (SD), median and interquartile range (IQR), parametric reference ranges (mean ± 2SD) and non-parametric reference ranges (2.5th–97.5th percentiles). Distributional normality was assessed using the Shapiro–Wilk test, reference ranges were defined using non-parametric 95% intervals. Values were considered abnormal if they fell outside this range (*p* < 0.05). Paired comparisons were performed to evaluate systematic differences between left and right ear responses at each intensity.

## 3 Results

The study population consisted of 32 Pomeranian dogs with CM (31 with CM1 and one dog with CM2) and 10 control Pomeranian dogs (CM0, Table 1). Some of the dogs presented with mild, owner-reported signs reported previously, including phantom scratching, vocalisation, head shaking, spontaneous signs of pain, and air licking (8). The CM dogs included 17 males and 15 females, ranging in age from 9 to 101 months (median 39 months), with body weights ranging from 0.9 to 6.4 kg (median 3.1 kg). The control dogs were predominantly male (70%), with ages ranging from 21 to 68 months (median 37 months) and body weights between 2.5 and 6.3 kg (median 3.9 kg).

**Table 1:** Example dataset of 2 dogs, dog 1 from the control group, and dog 2 from the CM group.

Dog	Group	Wave	Seconds	Ear	Age (months)	Sex	DB
1	Control	I	1.6	L	68	M	20
1	Control	I	2.3	R	68	M	20
1	Control	I	1.3	L	68	M	30
1	Control	I	1.6	R	68	M	30
1	Control	I	1.8	L	68	M	40
1	Control	I	1.8	R	68	M	40
1	Control	I	1.7	L	68	M	50
1	Control	I	1.8	R	68	M	50
1	Control	I	1.3	L	68	M	60
1	Control	I	1.8	R	68	M	60
1	Control	I	1.4	L	68	M	70
1	Control	I	1.7	R	68	M	70
1	Control	I	1.3	L	68	M	80
1	Control	I	1.3	R	68	M	80
1	Control	I	1.1	L	68	M	90
1	Control	I	0.9	R	68	M	90
1	Control	III	2.9	L	68	M	20
1	Control	III	3.6	R	68	M	20
1	Control	III	2.8	L	68	M	30
1	Control	III	3.7	R	68	M	30
1	Control	III	2.8	L	68	M	40
1	Control	III	3.3	R	68	M	40
1	Control	III	2.8	L	68	M	50
1	Control	III	2.9	R	68	M	50
1	Control	III	2.8	L	68	M	60
1	Control	III	2.8	R	68	M	60
1	Control	III	2.8	L	68	M	70
1	Control	III	2.6	R	68	M	70
1	Control	III	2.8	L	68	M	80
1	Control	III	2.6	R	68	M	80
1	Control	III	27	L	68	M	90
1	Control	III	3.1	R	68	M	90
1	Control	V	4.4	L	68	M	20
1	Control	V	4.3	R	68	M	20
1	Control	V	3	L	68	M	30
1	Control	V	4.4	R	68	M	30
1	Control	V	4.4	L	68	M	40
1	Control	V	3.9	R	68	M	40
1	Control	V	3.9	L	68	M	50
1	Control	V	3.5	R	68	M	50
1	Control	V	3.5	L	68	M	60
1	Control	V	3.6	R	68	M	60
1	Control	V	3.6	L	68	M	70
1	Control	V	3.7	R	68	M	70
1	Control	V	3.7	L	68	M	80
1	Control	V	3.8	R	68	M	80
1	Control	V	3.8	L	68	M	90
1	Control	V	3.8	R	68	M	90
1	Control	I–III	1.3	L	68	M	20
1	Control	I–III	1.3	R	68	M	20
1	Control	I–III	1.5	L	68	M	30
1	Control	I–III	2.1	R	68	M	30
1	Control	I–III	1	L	68	M	40
1	Control	I–III	1.5	R	68	M	40
1	Control	I–III	1.1	L	68	M	50
1	Control	I–III	1.1	R	68	M	50
1	Control	I–III	1.5	L	68	M	60
1	Control	I–III	1	R	68	M	60
1	Control	I–III	1.4	L	68	M	70
1	Control	I–III	0.9	R	68	M	70
1	Control	I–III	1.5	L	68	M	80
1	Control	I–III	1.3	R	68	M	80
1	Control	I–III	1.6	L	68	M	90
1	Control	I–III	2.2	R	68	M	90
1	Control	I–V	2.8	L	68	M	20
1	Control	I–V	2	R	68	M	20
1	Control	I–V	1.7	L	68	M	30
1	Control	I–V	2.8	R	68	M	30
1	Control	I–V	2.6	L	68	M	40
1	Control	I–V	2.1	R	68	M	40
1	Control	I–V	2.2	L	68	M	50
1	Control	I–V	1.7	R	68	M	50
1	Control	I–V	2.2	L	68	M	60
1	Control	I–V	1.8	R	68	M	60
1	Control	I–V	2.2	L	68	M	70
1	Control	I–V	2	R	68	M	70
1	Control	I–V	2.4	L	68	M	80
1	Control	I–V	2.5	R	68	M	80
1	Control	I–V	2.7	L	68	M	90
1	Control	I–V	2.9	R	68	M	90
1	Control	III–V	1.5	L	68	M	20
1	Control	III–V	0.7	R	68	M	20
1	Control	III–V	0.2	L	68	M	30
1	Control	III–V	0.7	R	68	M	30
1	Control	III–V	1.6	L	68	M	40
1	Control	III–V	0.6	R	68	M	40
1	Control	III–V	1.1	L	68	M	50
1	Control	III–V	0.6	R	68	M	50
1	Control	III–V	0.7	L	68	M	60
1	Control	III–V	0.8	R	68	M	60
1	Control	III–V	0.8	L	68	M	70
1	Control	III–V	1.1	R	68	M	70
1	Control	III–V	0.9	L	68	M	80
1	Control	III–V	1.2	R	68	M	80
1	Control	III–V	1.1	L	68	M	90
1	Control	III–V	0.7	R	68	M	90
2	CM	I	1.4	R	24	F	20
2	CM	I	1.1	R	24	F	30
2	CM	I	1.5	R	24	F	40
2	CM	I	1.8	R	24	F	50
2	CM	I	1.3	L	24	F	60
2	CM	I	1.4	R	24	F	60
2	CM	I	1.3	L	24	F	70
2	CM	I	1.5	R	24	F	70
2	CM	I	1.2	L	24	F	80
2	CM	I	1.4	R	24	F	80
2	CM	I	1.1	L	24	F	90
2	CM	I	1.3	R	24	F	90
2	CM	III	3.4	R	24	F	20
2	CM	III	3.3	R	24	F	30
2	CM	III	3.1	R	24	F	40
2	CM	III	2.9	R	24	F	50
2	CM	III	2.8	L	24	F	60
2	CM	III	2.8	R	24	F	60
2	CM	III	2.6	L	24	F	70
2	CM	III	2.8	R	24	F	70
2	CM	III	2.5	L	24	F	80
2	CM	III	2.7	R	24	F	80
2	CM	III	2.5	L	24	F	90
2	CM	III	2.7	R	24	F	90
2	CM	V	5.1	R	24	F	20
2	CM	V	4.6	R	24	F	30
2	CM	V	4.4	R	24	F	40
2	CM	V	4.4	R	24	F	50
2	CM	V	4.1	L	24	F	60
2	CM	V	3.9	R	24	F	60
2	CM	V	3.9	L	24	F	70
2	CM	V	3.7	R	24	F	70
2	CM	V	3.6	L	24	F	80
2	CM	V	3.5	R	24	F	80
2	CM	V	3.6	L	24	F	90
2	CM	V	3.7	R	24	F	90
2	CM	I–III	2	R	24	F	20
2	CM	I–III	2.2	R	24	F	30
2	CM	I–III	1.6	R	24	F	40
2	CM	I–III	1.1	R	24	F	50
2	CM	I–III	1.5	L	24	F	60
2	CM	I–III	1.4	R	24	F	60
2	CM	I–III	1.3	L	24	F	70
2	CM	I–III	1.3	R	24	F	70
2	CM	I–III	1.3	L	24	F	80
2	CM	I–III	1.3	R	24	F	80
2	CM	I–III	1.4	L	24	F	90
2	CM	I–III	1.4	R	24	F	90
2	CM	I–V	3.7	R	24	F	20
2	CM	I–V	3.5	R	24	F	30
2	CM	I–V	2.9	R	24	F	40
2	CM	I–V	2.6	R	24	F	50
2	CM	I–V	2.8	L	24	F	60
2	CM	I–V	2.5	R	24	F	60
2	CM	I–V	2.6	L	24	F	70
2	CM	I–V	2.2	R	24	F	70
2	CM	I–V	2.4	L	24	F	80
2	CM	I–V	2.1	R	24	F	80
2	CM	I–V	2.5	L	24	F	90
2	CM	I–V	2.4	R	24	F	90
2	CM	III–V	1.7	R	24	F	20
2	CM	III–V	1.3	R	24	F	30
2	CM	III–V	1.3	R	24	F	40
2	CM	III–V	1.5	R	24	F	50
2	CM	III–V	1.3	L	24	F	60
2	CM	III–V	1.1	R	24	F	60
2	CM	III–V	1.3	L	24	F	70
2	CM	III–V	0.9	R	24	F	70
2	CM	III–V	1.1	L	24	F	80
2	CM	III–V	0.8	R	24	F	80
2	CM	III–V	1.1	L	24	F	90
2	CM	III–V	1	R	24	F	90

DB, decibels; CM, Chiari Malformation; L, left; R, right; F, female; M, male.

Statistical testing using the Mann–Whitney U test confirmed no significant difference in age distribution (control: 37 months ((range, 21–68)); CM: 39 months ((range, 9–101)), *p* = 0.47) or body weight (control median body weight 3.9 kg and 3.1 kg in the CM group, *p* = 0.114) between the groups (Table 2). Body weight was not considered a relevant confounding variable for inclusion in the final linear mixed-effects model.

**Table 2:** Individual data of control and CM group.

Group	Age (months)	Sex	• Body weight (kg)
C	68	M	6.3
C	34	M	3.1
C	32	M	3
C	35	M	3.8
C	23	M	3.6
C	26	M	2.5
C	40	F	5
C	39	F	4.4
C	61	F	4
C	21	M	4.2
CM	24	F	2.4
CM	60	F	0.935
CM	60	F	3.1
CM	54	F	2.2
CM	53	M	6.3
CM	42	F	2.8
CM	56	F	5.6
CM	38	M	3.3
CM	39	F	3.5
CM	38	M	6.4
CM	34	M	3.8
CM	30	M	3.4
CM	36	F	4.35
CM	29	M	2.5
CM	25	MN	3.2
CM	101	F	3.5
CM	29	M	3.6
CM	10	M	2.8
CM	56	M	1.9
CM	59	M	6.3
CM	40	FN	1.9
CM	42	F	1.9
CM	22	M	1.6
CM	32	M	3
CM	52	FN	4.4
CM	44	F	3
CM	9	F	2.04
CM	39	F	2.2
CM	44	M	2.7
CM	23	M	3.4
CM	68	M	6.3
CM	39	M	5

F, female; FN, neutered female; M, male; MN, neutered male.

A linear mixed-effects model showed a significant difference between ears, with the right ear demonstrating longer latencies than the left ear (estimate = 0.047, *t* = 3.67, *p* < 0.001). There was no significant effect of group (CM versus control) on latency (*t* = −0.138, *p* = 0.891), and there was no significant interaction between group and ear (*t* = −0.033, *p* = 0.902).

## 4 Discussion

The brainstem auditory evoked response (BAER) is an assessment of auditory function. In animals, a behavioural reaction to noise stimuli is routinely tested during a standard neurological examination. This method allows for the determination of the presence or absence of hearing but does not allow for an objective assessment of the quality of hearing or partial hearing response [8]. The BAER response traces the signals from the auditory nerve through the brainstem to the auditory cortex and is recorded as electrophysiological signals (presented as peaks numbered I-VII) on an electrophysiological waveform. The height (amplitude) and duration of the peaks can be measured to assess normal hearing function [9, 10].

Cole et al. evaluated BAER latencies in Cavalier King Charles Spaniels with CM and noted variability among individuals and overlap with those of controls (21). That study lacked CM0 controls and examined differences between CM1 and CM2 dogs. They reported that CM2 dogs had longer wave latencies than CM1 dogs, but statistically significant differences were reported only for particular waves at particular signal intensities (such as wave V at 102 dB and wave II at 74 dB). They reported inconsistent interpeak latency intervals. Our findings included a CM0 control group, which allowed us to compare recordings of dogs with CM1 against a same-breed control, providing a more direct comparison between affected and unaffected dogs.

No significant differences were identified between CM1 and CM2 and control dogs for median latencies of waves I, III, and V or for the interpeak intervals I–III, I–V, and III–V in this study. These findings suggest that mild CM may not be sufficient to produce measurable abnormalities in BAER. The majority of affected dogs in this study were classified as CM1, with only one dog classified as CM2. It may therefore be assumed that mild morphological changes of the caudal cranial fossa do not substantially affect the auditory pathways, however, conclusions regarding more advanced, moderate-to-severe CM cannot be drawn from the present dataset.

In Pomeranians, CM has been associated with specific craniocervical morphometric abnormalities, including a shortened clivus and reduced caudal fossa dimensions measured on MRI and CT [9]. These structural changes may play a role in the pathogenesis of CM in the breed. However, the present findings suggest that such morphological abnormalities if present to a mild extent do not necessarily translate into detectable abnormalities in auditory brainstem conduction, at least in dogs with mild CM and classified as CM1.

In a cohort study of 200 human patients with Chiari malformation type I (CM1), BAER were found to be abnormal in 38.5% of cases. The most frequent alterations included a prolonged I–V interpeak interval and delayed wave V latency, observed in 31% of patients (22). Logistic regression analysis identified age, the degree of tonsillar ectopia, and clinical evidence of lower cranial nerve involvement as significant predictors of pathological BAERs.

Another group also identified conduction deficits in BAERs of CM1 and CM2 patients. Fifty-three of the 75 tested subjects had abnormalities, including prolongation of the III-V interpeak latency and prolongation of the I-V interpeak latency as well as an abnormal amplitude ratio V/I, smaller than 0.5 microvolts and absence of the wave V (23).

Di Stefano et al. examined BAERs and MRIs in human patients with intracranial hypotension, CM1 and sensorineural hearing loss and compared them with controls. Thirty-three % of the patients with CM1 showed abnormal BAERs, most notably a prolonged latency of wave V as well as extended I–III and III–V intervals (24).

The discrepancy between these human studies and the present findings may be explained by differences in disease severity and clinical status of the human patients. In the case of the latter, more severe CM, presence of tonsillar herniation and more advanced clinical neurological signs were reported (24). In contrast, the dogs in the present study were predominantly mildly affected. Although canine CM shares several pathophysiological features with human CM 1, there are interspecies differences. Human CM1 is primarily defined by cerebellar tonsillar herniation, whereas canine CM is associated with breed-related skull conformation, reduced caudal cranial fossa volume, cerebellar compression or vermis herniation, medullary kinking, and altered CSF flow dynamics (25, 26). These anatomical and biomechanical differences may influence the degree of brainstem involvement, including potential effects on auditory pathways (27). In human patients, auditory complaints or other neurological symptoms often prompt further diagnostic evaluation, allowing evoked potential findings to be interpreted in relation to clinical signs, disease severity, and sometimes surgical outcome during suboccipital decompression and duraplasty (26). In dogs, however, auditory dysfunction is more difficult to recognize clinically and cannot be assessed subjectively in the same way.

Breed-specific differences may also contribute to the discrepancy between the present findings and previous findings in dogs. CM in dogs is a multifactorial disorder affecting the skull, brain, craniocervical junction, and CSF circulation, and its morphology, prevalence and clinical relevance may differ between breeds. In CKCS, CM has a high prevalence of up to 99%, making it difficult to identify unaffected controls (28). In other breeds such as the American Griffon Bruxellois and Pomeranian, it has been reported to occur in 65 and 56% of the studied population, respectively (26) It remains unclear whether the morphological abnormalities at the craniocervical junction in various breeds translate into comparable and measurable auditory pathway dysfunction.

This study compared suprathreshold BAERs in a group of Pomeranians with CM versus a same-breed control group with defined reference ranges. Limitations include the relatively small cohort size and a lack of a separate group of dogs with CM2. Also, BAER wave amplitudes were not recorded in this study. Although body size may influence BAER latencies through differences in conduction distance, body weight did not differ significantly between groups in the present cohort. This interpretation assumes that body weight reliably reflects skull size, auditory pathway dimensions, and conduction distance, which may not necessarily be the case. It would be interesting to incorporate morphometric measurements, including caudal cranial fossa size in BAER recording analysis between dogs of one breed with various degrees of CM.

The present study focused on latency-based BAER parameters, including absolute wave latencies and interpeak intervals, because these measures are most directly related to auditory pathway conduction. BAER waveform amplitudes and amplitude ratios, such as the wave V/I amplitude ratio, were not evaluated. Although these parameters may provide additional information regarding the magnitude and synchrony of the neural response to auditory stimulation, their interpretation is more complex as it may be subject to inter- and intra-individual variability and may be influenced by factors such as electrode placement (29). Ideally, standardized amplitude reference values, including at least wave V amplitude and wave V/I ratio, could be established from a sufficiently large control cohort recorded under the same conditions to be able to compare with a CM-affected group. We found this beyond the scope of the current analysis. Hence, subtle abnormalities in amplitude or neural synchrony cannot be excluded.

A further limitation is that hearing thresholds were not determined. Threshold BAER testing requires repeated recordings at progressively decreasing stimulus intensities until the lowest reproducible waveforms are identified. This would have required a different and longer BAER protocol than the one used in this study. In addition, waveform interpretation could have been less reliable due to the smaller amplitude of the responses at low stimulus intensities. Hence, threshold assessment was considered beyond the scope of the original study design. As a result, subtle hearing impairment without detectable suprathreshold latency abnormalities cannot be excluded.

Future studies combining BAER with advanced morphometric and volumetric analyses would be interesting to determine whether electrophysiological abnormalities correlate with specific severity structural changes. It would be interesting to perform a longitudinal assessment to test whether BAER abnormalities occur at certain time points of clinical deterioration or with specific disease severity (dogs classified as CM2, for example) or MRI changes, thereby establishing their potential role as a biomarker of disease progression.

## Data Availability

The original contributions presented in the study are included in the article/supplementary material, further inquiries can be directed to the corresponding author.

## Footnotes

1
https://www.bva.co.uk/canine-health-schemes/cmsm-scheme/
